# Validation of an Automatic Arousal Detection Algorithm for Whole-Night Sleep EEG Recordings

**DOI:** 10.3390/clockssleep2030020

**Published:** 2020-07-16

**Authors:** Daphne Chylinski, Franziska Rudzik, Dorothée Coppieters ‘t Wallant, Martin Grignard, Nora Vandeleene, Maxime Van Egroo, Laurie Thiesse, Stig Solbach, Pierre Maquet, Christophe Phillips, Gilles Vandewalle, Christian Cajochen, Vincenzo Muto

**Affiliations:** 1 GIGA-Cyclotron Research Centre-In Vivo Imaging, University of Liège, Allée du 6 Août 8 B30, B-4000 Sart-Tilman, 4000 Liège, Belgium; daphne.chylinski@uliege.be (D.C.); mar.grignard@uliege.be (M.G.); nvandeleene@uliege.be (N.V.); maxime.vanegroo@uliege.be (M.V.E.); pmaquet@chuliege.be (P.M.); c.phillips@uliege.be (C.P.); gilles.vandewalle@uliege.be (G.V.); 2 Centre for Chronobiology, Psychiatric Hospital of the University of Basel, Willhelm Klein-Strasse 27, 4002 Basel, Switzerland; franziska.rudzik@posteo.de (F.R.); thiesse.laurie@gmail.com (L.T.); stig.solbach@unibas.ch (S.S.); christian.cajochen@upk.ch (C.C.); 3 Transfaculty Research Platform Molecular and Cognitive Neurosciences, University of Basel, Birmannsgasse 8, CHF-4055 Basel, Switzerland; 4 Department of Electrical Engineering and Computer Science, University of Liège, Allée de la Découverte 10 B28, B-4000 Sart-Tilman, 4000 Liège, Belgium; d.coppieters@uliege.be; 5 Department of Neurology, University of Liège Hospital, B35, B-4000 Liège, Belgium; 6 GIGA-In Silico Medicine, University of Liège, Avenue de l’Hôpital 1-11, B-4000 Liège, Belgium

**Keywords:** arousals, electroencephalography, automatic detection, sleep, artefacts

## Abstract

Arousals during sleep are transient accelerations of the EEG signal, considered to reflect sleep perturbations associated with poorer sleep quality. They are typically detected by visual inspection, which is time consuming, subjective, and prevents good comparability across scorers, studies and research centres. We developed a fully automatic algorithm which aims at detecting artefact and arousal events in whole-night EEG recordings, based on time-frequency analysis with adapted thresholds derived from individual data. We ran an automated detection of arousals over 35 sleep EEG recordings in healthy young and older individuals and compared it against human visual detection from two research centres with the aim to evaluate the algorithm performance. Comparison across human scorers revealed a high variability in the number of detected arousals, which was always lower than the number detected automatically. Despite indexing more events, automatic detection showed high agreement with human detection as reflected by its correlation with human raters and very good Cohen’s kappa values. Finally, the sex of participants and sleep stage did not influence performance, while age may impact automatic detection, depending on the human rater considered as gold standard. We propose our freely available algorithm as a reliable and time-sparing alternative to visual detection of arousals.

## 1. Introduction

Sleep is a complex phenomenon constituted of alternating stages that are characterised by inhomogeneous patterns of neuronal activity. Arousals during sleep consist in transient accelerations of the electroencephalogram (EEG) entwined with the structure of the sleep EEG [1]. Arousals are physiological components of sleep microstructure, thought to ensure the reversibility of sleep [2], which can be triggered by exogenous or endogenous stimuli. They are seen in increased amounts in some sleep pathologies, such as sleep apnoea, where they are concomitant with hypoxic events [3]. Arousals can be triggered by noise and their density is enhanced in noisy environments [4,5,6]. Spontaneous arousals (i.e., not associated with exogenous nor by detectable endogenous stimuli, such as hypoxia) are present in normal, non-pathological sleep, and have been reported to increase with age even though they are present at all ages [7,8]. Overall, arousals may lead to shallower sleep stages and contribute to more fragmented sleep.

Throughout the years, arousals have been defined in various ways, though the most widely accepted definition was proposed by the American Sleep Disorders Association (ASDA) in 1992 [9] and maintained later in American Academy of Sleep Medicine (AASM) publications [10,11,12]. They are described as abrupt and transient shifts in EEG frequencies that last at least 3 s and may include theta and alpha frequencies and frequencies greater than 16 Hz (but not spindles) and must be preceded by at least 10 s of stable sleep. Thus, a minimum of 10 s of stable sleep separates two distinct arousals. In Rapid Eye-Movement (REM) sleep, arousals must be accompanied by an increase in submental electromyogram (EMG) amplitude for at least 1 s. In general, arousal scoring cannot, however, be based solely on changes in submental EMG but must involve the aforementioned changes in EEG frequency. Importantly, arousal detection can affect sleep staging. According to AASM rules [10], once detected, an arousal may imply that the next 30 s window of sleep should be scored in another (lighter) sleep stage (e.g., N3 to N2/N1/wake, N2 to N1/wake, REM to N1/wake) unless the sleep features present in the next 30 s window plead to maintain the current sleep stage (e.g., presence of rapid eye movements in REM, or of k-complexes/spindles in N2). In addition, researchers favour the exclusion of artefacts and arousals from spectral decompositions of the sleep EEG signal in order to quantify artefact-free EEG power in different frequency bands.

Detecting arousals is, therefore, an important step in characterising sleep in clinical practice and in research settings. Nowadays, the detection of arousal events is commonly done by human visual inspection of the recordings. This method has the disadvantage of being time consuming and driven by a subjective interpretation of an “abrupt shift in EEG”, which may vary considerably among individual raters. Indeed, as for sleep stages or spindle scoring [13,14], arousal scoring is subject to intra-rater variability (as scorers would not necessarily detect events consistently if presented with the same recording twice [15]), as well as inter-rater variability. The literature shows that inter-rater variability differs from study to study. It may be hard to compare them, given the heterogeneity in methodology or agreement coefficients used, but what stands out is that while inter-rater agreement can be good when comparing visual scorers from the same centre (intraclass correlation coefficient 0.84 [16], 0.90 [17], event by event agreement 90% [18], Cohen’s kappa 0.71 [19]), it proves lower when comparing arousal scoring across different centres [20], when scoring is based solely on EEG traces without autonomic responses (heart rate or airflow) [18], or when considering only light sleep periods [15].

As for sleep staging, where the importance of automatic methods is continuously growing [21,22], automatic arousal detection represents a way of getting around these difficulties and increasing reproducibility. In the past few years, several automated algorithms for arousal detection, based on EEG scoring have been proposed [23,24,25,26,27,28]. However, most of them are based on some form of deep learning and require a training phase [29,30], use Independent Component Analyses (ICA) [31], or spatial and temporal features, and their implementation remains largely user-dependent, or they have not been validated using a comprehensive set of agreement measures. Usually, automatic detection methods used for transitory events (e.g., sleep spindles) yield a higher count of events than visual detection [32,33].

Here, we adapted a validated and published automatic artefacts and arousals detection algorithm [34], which was developed as a method to detect the clean segments in EEG data in order to subsequently perform spectral power analysis. We aimed to separate its detection into both artefacts and arousals to offer a quantification tool for finer gradient analysis of arousals, which are meaningful events in the sleep EEG. A key characteristic of the algorithm is that detection thresholds are self-adjusting to individual recording features. We validated the arousals detection on a dataset composed of 35 undisturbed younger (N = 18; age 24 ± 3 y) and older (N = 17; age 61 ± 6 y) participants’ night-sleep EEG recordings. They were first visually scored for sleep stages and arousals by four sleep expert human raters (HR) from Basel, Switzerland, and considered as a single rater (indicated through the manuscript as BAS). Arousals were then visually scored by another expert from Liège, Belgium (here defined as DC, first author) who had access to partial information about sleep stages (wake-NREM sleep-REM sleep) to avoid potential bias arising from sleep stage changes. Arousals were also automatically detected (AD). We computed several agreement coefficients: inter-rater agreement (S), Cohen’s kappa (κ), sensitivity (Se), mean overlap of detected events (C), and false discovery ratio (FDR), considering different references or gold standard detection (see methods, Section 2.4.1 for coefficient definitions and computation). We further explored whether sex, age, and sleep-stage influence detection reliability. Our objective was to demonstrate that the algorithm is a reliable arousal detection method, which gives similar agreement performances as human raters among themselves. Moreover, we expected more arousals to be AD, based on previous automatic detection methods for transitory events, and that supplemental arousals would contain lower frequency oscillation—rendering them less obvious to the human eye.

## 2. Materials and Methods

### 2.1. Dataset

This is a retrospective study, taking advantage of data collected and published elsewhere [6]. Our dataset consisted of 35 whole-night multichannel EEG recordings of undisturbed sleep in younger (age range: 19–29, µ = 24.07 ± 3, N = 18, 7 females) and older (age range: 51–70, µ = 61.38 ± 6, N = 17, 8 females) participants, randomly selected to have matching group sizes. Participants had good self-reported sleep quality, as assessed by the Pittsburgh Sleep Quality Questionnaire [35] (PSQI ≤ 5), normal levels of daytime sleepiness, as assessed by the Epworth Sleepiness Scale [36] (ESS ≤ 10), and did not present periodic limb movement disorder nor sleep-disordered breathing, as verified during a polysomnography prior to inclusion in the study. The recordings contained 12 EEG derivations placed according to the 10–20 system (F3, Fz, F4, C3, Cz, C4, P3, Pz, P4, O1, Oz, O2), referenced against the average of the two mastoids, as well as submental electromyogram (EMG) and electrooculogram (EOG) bipolar channels, all collected via a Vitaport-3 digital recorder (TEMEC Instruments B.V., Kerkrade, The Netherlands) at an acquisition sampling rate of 256 Hz and a storage sampling rate of 128 Hz. Signals were filtered at acquisition (between 0.159 and 30 Hz for the EEG/EOG and between 1 and 70 Hz for EMG).

### 2.2. Human Arousals Scoring

Recordings were first randomly assigned to one of four human raters (HR) in Basel (BAS1-4)—Table 1 shows the distribution across raters—and scored for sleep stages and arousals according to standard criteria [37]. Importantly, the BAS scorers were not blind to recordings’ age group. The recordings were further visually scored for arousals by a second HR (DC), who had access to sleep staging information only in the form of NREM/REM/wake epochs, to avoid biasing that would incline to detect arousals in the case of a sleep change to a lighter NREM sleep stage, and was blind to participants’ age group. A supplementary analysis with Kruskal–Wallis test for unequal samples showed no significant differences in Cohen’s κ values between the different BAS scorers when using DC as gold standard (Chi-square = 2.51, *p* = 0.47, df = 3), nor when using BAS as gold standard (Chi-square = 3.36, *p* = 0.34, df = 3). BAS scorers were, therefore, all considered as one scorer for the remaining analyses.

### 2.3. Automatic Arousals Detection Algorithm

For the automatic detection procedure, we adapted an algorithm initially developed for artefact detection (i.e., both true artefacts and arousals) which is based on time and frequency analysis with adapted thresholds derived from the data [34]. The method consists in successively applying several modules, which are briefly described hereafter. Modifications done to the original code consisted only in the organisation and extraction of arousal events. More detailed information about signal processing can be found in [34].

#### 2.3.1. Preprocessing

Recordings were first filtered (Butterworth filter of order 3; high pass/low pass at 0.5/30 Hz for EEG; 0.1/5 Hz for EOG; 10/100 Hz for EMG) and considered in scoring windows of 30s, then partitioned into 1s epochs. The signal of each channel was further mean corrected.

#### 2.3.2. Bad Channel Detection

EEG: Obvious bad channels (i.e., flat and noisy channels with a signal standard deviation (SD) lower than 1 µV or higher than 6.10^3^ µV, respectively, were detected over the entire recording. Then, a finer detection was performed over each 30 s window using each channel’s SD and comparing it to the SD of other (good) channels—a channel with a deviating ratio >5 was marked as a bad EEG channel.

EMG: As the EMG signal is typically displayed in a bipolar montage but can inconsistently contain artefacts solely on one of the two channels, the module attempted to reconstruct usable data from the available EMG channels. Flat channels were detected per 30 s window as EMG channels referenced to the montage reference displaying median value <0.1 µV. If no channel was considered flat, noisy channel detection was performed per 30 s window by identifying referenced channels for which the median was at least twice that of the other. Noisy channels were then tested to differentiate constant from transitory noise, considering all 1 s epochs in a given 30 s window. A channel was noisy over a 1 s epoch if the ratio of absolute mean values of both EMG channels was larger than 2, and noisy on a whole scoring window if more than half of the epochs (>15 s) were noisy. A composite EMG channel was finally reconstructed based on the remaining (clean) EMG signal arising from both or only one EMG channel(s).

#### 2.3.3. Features Extraction

Shift in EMG: Transient increases in muscular tone frequency/magnitude were detected in three steps. First, the rejection of the highest abnormal activities was performed along the whole EMG channel by identifying any recording period that reached higher values than baseline EMG values extracted from the first four minutes of the recording, which were considered to be the highest muscular tone amplitude without artefact. A second step then detected peaks in EMG activity in shorter time windows in order to account for EMG background activity, by defining, for each scoring window, a specific threshold as being the output of a median filter applied on a symmetric-centred 3-s scoring window. A last step aimed at identifying the relevant EMG peaks that could influence EEG, by assessing their intensity and duration and comparing them to an adapting threshold computed based on the first ten 1 s epochs on both sides of the muscle tone peak.

Shift in EEG: In this step, two tests were performed over three frequency bands: alpha (7–13 Hz), beta (16–30 Hz), and theta (3–7 Hz). First, the detection of abnormal EEG activity was carried out using a fixed threshold consisting in the median value of the power in the given frequency band (α, β, θ) of the whole recording. A second step took into account the specific background EEG activity of a shorter time window. For each scoring window, all 1 s epochs without a corresponding EMG shift were selected, plus the first ten 1 s epochs without an EMG shift each side of that scoring window. For the three examined frequency bands, all shifts in EEG higher than the adapted threshold, corresponding to twice the median value of the selected epochs, were considered. The 1 s epochs considered as containing a spindle were computed as those with a relative power in the sigma band (i.e., 11–16 Hz) higher than 85% of the maximum relative power in the sigma band over the entire recording. The relative power in the sigma band was computed as the ratio of the sigma band power over the sum of power in alpha, sigma and beta bands.

#### 2.3.4. Arousal Detection

Finally, shifts in EEG in the α, β, or θ frequency band (but excluding those concomitant with a shift in the sigma band) lasting over at least three consecutive 1 s epochs were selected. These were further checked for muscular tone increase to be classified as EMG-associated events. In REM, only events with a concomitant EMG tone increase were considered as arousals.

### 2.4. Comparison between Raters

As automatic detection (AD) reports events in seconds, HR scores were converted into 1 s resolution: any 1 s epoch containing more than 0.5 s marked as arousal was labelled as containing an arousal. To compute agreement coefficients, one must compare the detection of a rater against another that is considered as the gold standard. For comparison between HR, as there is no way to know which events are “true arousals”, the detection of each HR was compared against the detection of the other HR in turn. To compare AD to HR, two HR detections were created: an inclusive detection (HR inclusive), with all arousals found by HR (no matter if they were identified only by one HR, or by both); a conservative detection (HR conservative), with only those arousals that were identified by both HR. As it is possible that the detection of a frequency shift in EEG as an arousals is more likely in the presence of a concomitant EMG tone increase, the analyses considered all arousals found by the AD, as well as those found by AD that were associated with an EMG tone increase (AD EMG).

**Figure 1:**
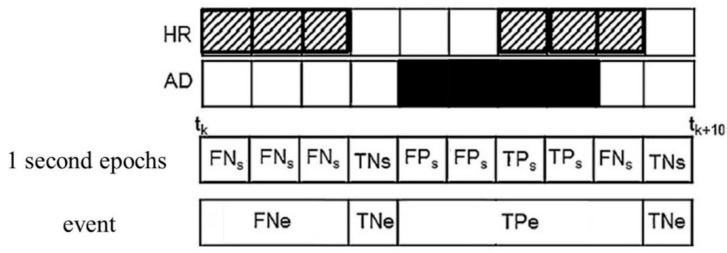
Two types of comparison level made—1 s epoch or event. Gold standard human rater (HR) scoring is represented on the top line, with arousals marked by the hatched squares. Automatic Detection (AD) is on the second line with black squares marking detected events. Adapted from [34]. True positive (TP): 1 s epoch/event marked as arousal both by AD and HR. False positive (FP): 1 s epoch/event marked as arousal by AD but not by HR. True negative (TN): 1 s epoch/event marked as arousal-free by both AD and HR. False negative (FN): 1 s epoch/event marked as arousal free by AD but as an arousal by HR.

Two types of comparison were made with HR as gold standard—at the level of a 1 s epoch and at the event level (i.e., the set of consecutive 1 s epochs forming an arousal), where events were considered as common between HR and AD if there was at least 1 s of the event overlapping across raters. Each comparison received one of the following labels shown in Figure 1.

#### 2.4.1. Statistical Parameters

We computed agreement with several coefficients classically found in the literature. At the 1 s epoch level, we computed two values, the inter-rater agreement S (Bennett et al., 1954) and Cohen’s Kappa (κ) (Cohen, 1960), following the equations below. Although inter-rater agreement S does not account for the unbalanced nature of the data to compare (the ratio between epochs containing arousals and those who do not is usually around 5 to 95%) and tends, thus, to overestimate inter-rater concordance, it is amongst the most commonly used. Cohen’s Kappa, in contrast, takes into account the unbalanced scores but is frequently judged overly conservative. The interpretation of κ values can be seen in Table 2 (from [38]).

(1) $$S_{s}=2\times P_{0}-1\;where\;P_{0}=\frac{{\mathrm{TP}}_{s}+{\mathrm{TN}}_{s}}{{\mathrm{TP}}_{s}+{\mathrm{TN}}_{s}+{\mathrm{FP}}_{s}+{\mathrm{FN}}_{s}}$$

(2) $$\kappa_{s}=\frac{P_{0}-P_{r}\;}{1-P_{r}}\;where\;P_{r}=\frac{\left({\mathrm{TP}}_{s}+{\mathrm{TN}}_{s}\right)\times \left({\mathrm{TP}}_{s}+{\mathrm{FP}}_{s}\right)}{{\left({\mathrm{TP}}_{s}+{\mathrm{TN}}_{s}+{\mathrm{FP}}_{s}+{\mathrm{FN}}_{s}\right)}^{2}}$$

At the event level, we calculated inter-rater agreement via the three following criteria: sensitivity in terms of the percentage of detected event (Se); averaged overlap of the detected events (C); false discovery ratio (FDR). Sensitivity gives an idea of how well the AD detects events that are detected by the gold standard; C is the mean proportion of TP_s_ contained in TP_e_ (delimited by the HR’s detection); FDR corresponds to the proportion of false detections to the total number of detections.

#### 2.4.2. Time Frequency Analysis

As AD detected many more arousals than HR, we wanted to further characterise the events that were not visually detected. For this analysis we took into account only the first three seconds of each arousal, considering that, by definition, an arousal lasts a minimum of 3 s. We performed a time-frequency analysis of all AD arousals using Morlet’s method [39] with 1 Hz bins, after performing a baseline correction on the first 500 ms. We summed the power in each 1 Hz bin for the 3 s duration and computed their relative power by dividing the power in each bin over the power in all frequency bins (0.5–29.5 Hz). We then computed the sum of the relative power in the theta (4.5–7.5 Hz), alpha (8.5–11.5 Hz), and beta (16.5–29.5 Hz) bands.

#### 2.4.3. Statistical Analyses

Statistical analyses were performed in SAS 9.4 (SAS Institute, Cary, NC). For generalised linear mixed models (GLMM), the distribution of dependent variables was first determined in MATLAB using the allfitdist function (developed by Mike Sheppard), and the models were appropriately adjusted. The subject was put as a random factor. Statistical significance was set at *p* < 0.05. Degrees of freedom were estimated using Kenward–Roger’s correction. *p*-values in post-hoc contrasts (difference of least square means) were adjusted for multiple testing using Tukey’s procedure.

## 3. Results

### 3.1. Comparison of Human Raters (HR)

**Figure 2:**
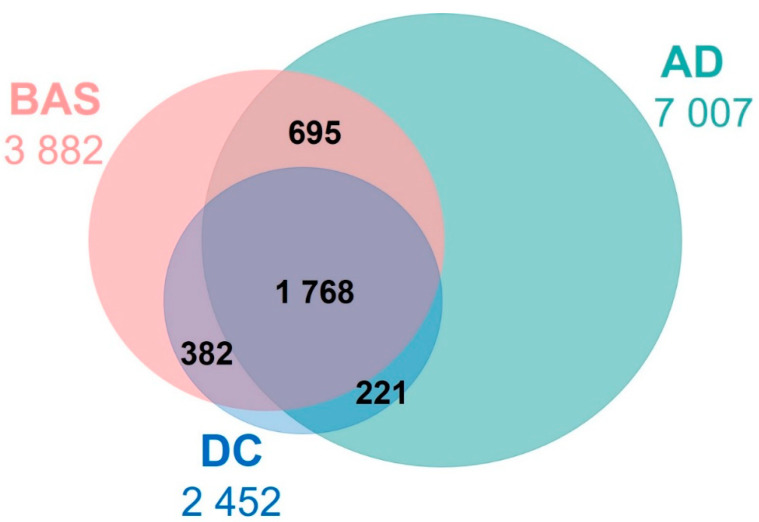
Total number of detected arousal events over all 35 recordings for Basel HR (BAS), Liège HR (DC), and automatic detection (AD), as well as their overlap.

Comparing detections can only be done if a “ground truth” or suitable approximation (i.e., an expert HR as gold standard) is used as reference. As a first step, visual arousal detections were compared across HR, using each HR as gold standard in two separate analyses, as shown in Table 3. Agreement was high in both analyses, with inter-rater agreement S and Cohen’s κ values indicating very good agreement (Table 2 in Section 2.4.1. shows Cohen’s kappa values and interpretations, from [38]), so that we considered both detections as good. BAS detected more arousals than DC, as shown in Figure 2, which was reflected by a relatively low sensitivity when using BAS as gold standard and a high FDR when using DC as gold standard. In general, arousals detected by DC were also detected by BAS, as reflected by a low FDR when using BAS as gold standard and a high sensitivity when using DC as gold standard. Commonly detected arousal events were well agreed between raters with about 75% of 1 s epochs overlapping for each detected event (C).

### 3.2. Automatic Arousal Detection (AD) vs. Human Raters

**Figure 3:**
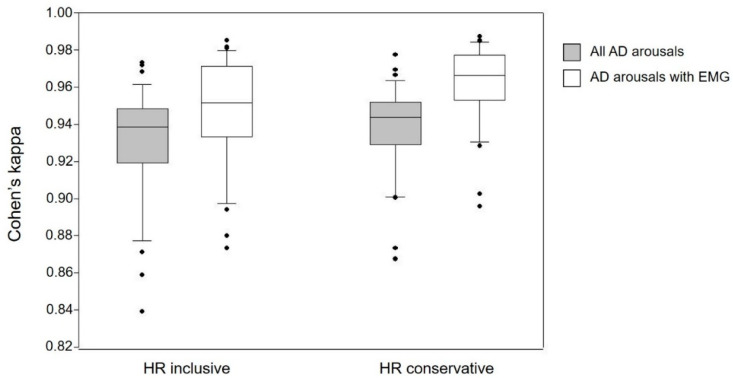
Box plot of Cohen’s kappa values for HR inclusive and HR conservative as gold standard (values for all AD arousals in grey, for EMG-associated AD only in white). The boxes’ central lines indicate the medians of κ values, with the bottom and upper edges showing the 25th and 75th percentiles, respectively. The whiskers extend to the most extreme data points not considered outliers—outliers were not removed from the plot.

We then computed agreement coefficients for AD by comparing it against two gold standard HR detections—either including all the arousals found by either of the HR (HR inclusive) or only those common to both HR (HR conservative), as shown in Table 4. Agreement was high, with κ values indicating very good agreement, and inter-rater agreement S was very good (>85%) when using both HR inclusive and HR conservative as gold standard, as shown in Figure 3. Similar to HR comparison, commonly detected arousal events were well agreed between raters, with about 60% of 1 s epochs overlapping for both HR inclusive and HR conservative as gold standard. As expected, AD detected many more arousals than HR, as shown in Figure 2 (1.8-times more than BAS and 2.9-times more than DC), with high FDR values, particularly for HR conservative, which is to be expected as this detection comprises substantially less events. Considering only AD arousals associated with a concomitant EMG tone increase substantially improves FDR, with little impact on indices S, κ, and C, but, expectedly, reduces Se.

#### 3.2.1. Impact of Age and Sex

We checked whether recordings of older individuals bear more arousals than recordings of younger individuals. Two sample *t*-tests revealed no such significant difference, except for Basel scoring (*t* (125.11) = −3.95, *p* = 0.0006), as shown in Table 5.

Generalised Linear Mixed Models (GLMMs), with each of the agreement coefficients as the dependent variable in turn, showed that, for AD against HR inclusive, there was a significant effect of age on sensitivity and FDR, with higher values for both in the young group, as shown in Table 6. For events automatically detected with a concomitant submental EMG tone increase against HR inclusive as gold standard, significantly higher values were found in the younger age group for inter-rater agreement, Cohen’s κ, and sensitivity.

When comparing AD against HR conservative, no significant effect of age nor sex was found. When taking into account only AD events with concomitant EMG tone increase, higher sensitivity for the younger age group was found, as well as a slightly higher inter-rater agreement and Cohen’s κ for female recordings.

Given the high number of tests performed, a corrected *p* value threshold was set at *p* = 0.005 (Bonferroni correction for 10 comparisons; for each HR detection (inclusive/conservative) 5 coefficients ×2 comparison levels—all AD and AD EMG). Only inter-rater agreement S and Cohen’s κ for HR inclusive, when taking into account AD EMG events, meet the correction criteria.

In summary, while the sex of the recorded participant did not seem to influence the agreement between AD and HR, age may impact agreement, particularly when considering HR inclusive as gold standard and when considering only EMG-associated AD events.

#### 3.2.2. Impact of Sleep Stage

**Figure 4:**
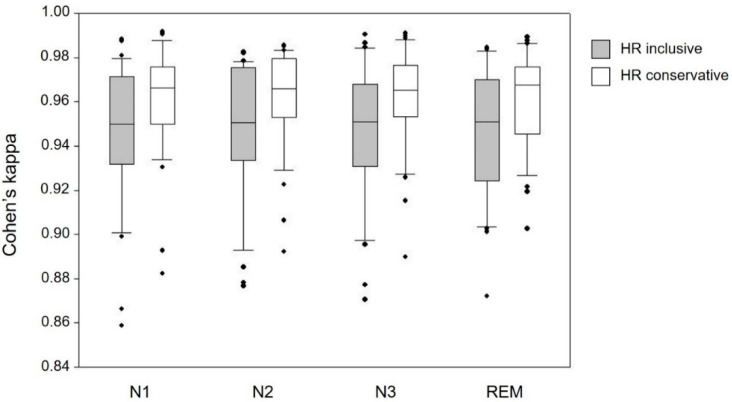
Box plot of Cohen’s kappa values for HR inclusive and conservative detection as gold standard by sleep stage. The boxes’ central lines indicate the medians of κ values, with the bottom and upper edges showing the 25th and 75th percentiles, respectively. The whiskers extend to the most extreme data points not considered outliers, and outliers were not removed from the plot.

As not all types of events (true positive, true negative, false positive and false negative) were present for all sleep stages, comparison coefficients based on events (rather than seconds) were not suitable for testing differences in agreement across sleep stages (see methods, Section 2.4 for coefficient computation). Hence, arousal detection over different sleep stages was compared only using comparison coefficients based on 1 s epochs (i.e., Cohen’s κ and inter-rater agreement S). For comparison of AD against HR inclusive, a GLMM with Cohen’s κ as dependent variable showed no significant effect of age (F(1,31.43) = 2.24, *p* = 0.15), sex (F(1,31.53) = 2.91, *p* = 0.10) or sleep stage (F(3,72.55) = 0.42, *p* = 0.74). Likewise, another GLMM with inter-rater agreement S showed no significant effect of any of the three variables (age F(11.59) = 2.55, *p* = 0.12; sex F(1,31.68) = 3.01, *p* = 0.09; sleep stage F(3,72.82) = 0.43, *p* = 0.73) Another set of GLMMs of AD against HR conservative showed no significant effect of age (F(1,31.69) = 0.02, *p* = 0.88), sex (F(1,31.77) = 1.66., *p* = 0.21), nor sleep stage (F(3,72.29) = 0.08, *p* = 0.97). As for inter-rater agreement, S, again, no significant effect of age (F(1,31.77) = 0.01, *p* = 0.93), sex (F(1,31.84) = 1.7, *p* = 0.20) or sleep stage (F(3,72.53) = 0.09, *p* = 0.97) was found. Figure 4 shows the distribution of Cohen’s κ values across sleep stages for HR inclusive and conservative as gold standard.

These results indicate that, when taking into account the potential variability in AD performance, according to sleep stages, agreement between AD and HR does not depend on age, sex, or sleep stage.

#### 3.2.3. Correlation between AD and HR

**Figure 5:**
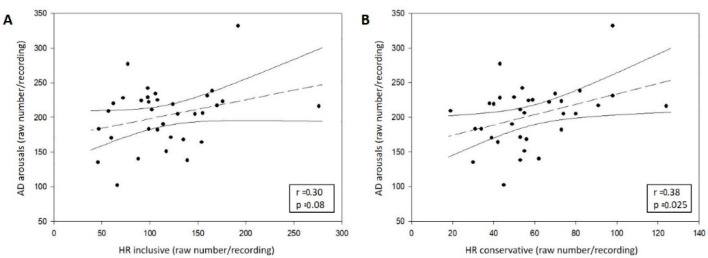
Correlation between all AD arousals and HR for inclusive (**A**) and conservative (**B**) detection.

The next question we asked beyond these agreement estimations was whether AD and HR were correlated (i.e., whether variation in arousal density across recordings were similarly detected by HR and AD). Pearson r indicated that the correlation between AD and HR inclusive was r = 0.30 (*p* = 0.08) and r = 0.23 (*p* = 0.19) for all AD events, as shown in Figure 5 in panel A, and AD EMG-associated events, respectively. For HR conservative, the correlation was r = 0.38 (*p* = 0.025) and 0.36 (*p* = 0.037) for all AD events, as shown in Figure 5 in panel B, and AD EMG-associated events, respectively. It seems, therefore, that AD is best associated with the most conservative detection of arousals across centres.

### 3.3. Characterisation of the Arousals Only Detected by AD

As AD detected many more arousals than HR, we wanted to further characterise the events that were not visually detected by any HR but were still picked up by AD, to see if they differed in terms of frequency or power. To do so, we performed a time-frequency analysis on the first 3 s of all AD arousals, in the frequencies 0.5 Hz–29.5 Hz by 1 Hz bins. We summed over time the power in each 1 Hz bin and normalised it by dividing by all frequency bins to obtain relative power. We then computed the sum of the relative power in the theta (4.5–7.5 Hz), alpha (8.5–11.5 Hz), and beta (16.5–29.5 Hz) bands.

**Figure 6:**
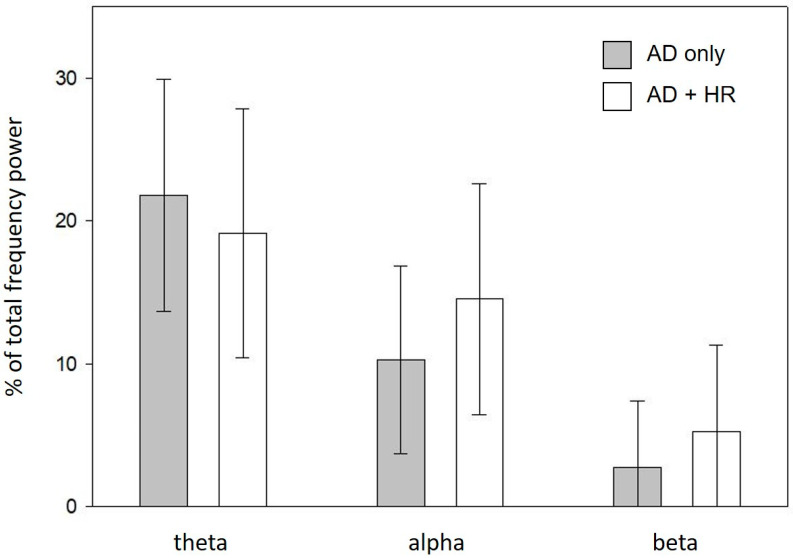
Mean relative frequency power for the theta, alpha, and beta bands for arousals detected only by AD (grey), and those detected by AD and HR (white). Error bars show standard deviation.

A GLMM with relative power as dependent variable showed main effects of frequency band (F(221,101) = 9991.38, *p* < 0.0001) and rater (F(121,101) = 296.47, *p* < 0.0001) and an interaction between frequency band and rater (F(221,101) = 473.23, *p* < 0.0001). Post hoc analysis showed the theta band had a significantly lower power (*p* < 0.0001), while the alpha and beta a significantly higher power (*p* < 0.0001) for AD arousals, that were also detected by an HR. Figure 6 shows mean relative frequency for the theta, alpha, and beta band for arousals that were detected only by AD and those detected by AD and HR.

## 4. Discussion

Arousals are transient changes in neuronal activity that are part of normal sleep microstructure and are reflected in the temporary acceleration of the EEG [9]. They are either spontaneous and show an increased prevalence with age, or are triggered by internal or external events [40]. Their detection is important for sleep stage determination and spectral composition analyses, as well as pathology characterisations, as they are considered to reflect poorer sleep quality. Here, we validate an automatic detection (AD) of spontaneous arousals using non-pathological sleep EEG recordings and human visual detection in two research centres as reference (Centre for Chronobiology in Basel, Switzerland, and GIGA-Cyclotron Research Centre-In Vivo Imaging in Liège, Belgium). AD was adapted based on a previously validated tool for the undifferentiated detection of both artefacts and arousals [34]. Our analyses first confirm that the number of spontaneous arousals visually detected by human raters (HR) can vary a lot, with sensitivity and FDR that can be, respectively, lower and higher, depending on which rater was considered as the gold standard [15]. As reported previously [15,20], differences in detection seem to arise from research centre “traditions/habits”, since considering the four different raters of Basel separately (vs. Liège) did not yield a different picture. Qualitative inspection of the data did not lead to other indications on the origin of the discrepancy between research centres. Despite these discrepancies, Cohen’s κ and inter-rater agreement S are high across HR (and, therefore, research centres) so that we considered both HR as equally good.

Critically, our results show that AD performed at least equally as well as HR when comparing against a composite detection of both HR. As it was not possible to objectively select, among visually detected arousals, those that would be the actual “true” arousals, composite detection was either inclusive (HR inclusive), with all arousals detected by either (or both) HR, or conservative (HR conservative), retaining only those “salient” arousals, common to both HR. The inter-rater agreement measure S (most commonly used, though not suitable for unbalanced data) and Cohen’s κ (more conservative but better suited) were always both in the upper ranges, indicating high agreement levels. AD detects most of what the HR detect, but also picks up many more arousals, as reflected by intermediate sensitivity and FDR levels. This is also noticeable for the automatic detection of sleep spindles, which are also transient accelerations of EEG intertwined in the rest of the EEG oscillations [32,33]. These additional arousals correspond to our mathematic translation of the AASM definition of an arousal [10], so they bear the characteristics of an arousal but their visual detection would be difficult, if at all possible. This statement is supported by the fact that arousals that were only detected automatically had lower relative power in the alpha and beta bands and higher power in the theta band. Increases in the theta band can, indeed, arguably be harder to disentangle from sleep background activity. This raises the question of what defines an arousal. Can arousals only be detected visually and, therefore, should one accept all the subjectivity and inter-rater variations? Should one keep on spending a lot of time detecting arousals in a manner that is not outperforming an automatic tool? We consider that AD has a clear added value as it spares time and increases reproducibility through an algorithmic definition of an arousal. In contrast to visual detection, it will always yield the same detection when using the same dataset and its detection bias will be systematic across research centre and study sample. Based on the current definition of an arousal, where, for instance, there is no objective threshold determining how large the frequency shift must be for an event to be classified as an arousal, there is no reason to consider that events that are only detected by the AD algorithm do not constitute arousals.

Although AASM recommendations state that arousals during NREM sleep do not need to be associated with changes in EMG tone [9], they might be easier to detect visually if immediately preceded or concomitant to muscular activity change. When we only considered arousals with EMG changes, AD detected far less arousals (from 200 ± 43 to 69 ± 23), as shown in Table 5, which leads to FDR levels comparable to inter-HR agreements. This positive effect is counterbalanced by an inevitable reduction in sensitivity, since there are fewer detected events. One is, therefore, facing two options when using our algorithm—either considering all detected arousals, even though a large part would not be visually detected, or considering only those that are associated with EMG changes, and acknowledging that a larger part of what would be visually detected is missing. Importantly, however, we found that AD and HR arousal densities over the whole night are correlated, particularly when using HR conservative as gold standard, meaning that an individual with more arousals following visual inspection will also have a lot of AD arousals, relative to other individuals of a sample. This tells us that AD can be used to characterise arousals in a recording of a dataset and compute correlations with other variables of interest (e.g., subjective sleep quality measures, cognitive assessments, genetic background, etc.). In addition, our dataset included 35 recordings of individuals of both sexes almost equally split between younger (<30 y) and older (50–70 y) adults. Although we did find differences in some agreement coefficients according to age, particularly when comparing against HR inclusive, we consider that this is likely due to the difference between age groups in the amount of arousals detected by BAS, as arousal density was similar across age-groups when using AD, as well as for Liège HR. The origin of the discrepancy regarding age between BAS, on the one hand, and DC and AD on the other, is unclear but may be related in part to the slight difference in methodology, where BAS raters had access to the age of the participants, together with their sex and to the full visual sleep staging (vs. NREM, REM, WAKE for DC). Crucially, sleep stage did not affect detection quality.

Other automatic detection tools of arousals are available. Some are based on ICA [31] but retain a subjective component in the selection of the principal components to attribute to arousals. In addition, the ICA tools do not allow us to count arousals, but rather to remove their bias in the processing of the data. Tools using temporal and spectral aspect, such as our algorithm, are also available. Some were only confronted to a single expert rating or ratings from a single research centre [23,24,25,26]. In addition, previous studies rely on agreement measures which are inappropriate for the detection of unbalanced events; i.e., where an event (e.g., arousal event) is much less represented compared to another (non-arousal event). In our view, the use of the more conservative Cohen’s κ thus strengthens the message from the present study.

Although constituting an important aspect of the sleep EEG, the current definition of arousals does not allow for a clear consensus on their visual detection, as shown by variable detections across HR and research centres. Here, we provide a novel arousal detection tool, which comes in addition to these previous tools and reaches agreement levels with HR comparable to HR among themselves. It has the advantage of not requiring a training phase or supervision and it is a fast and objective method of arousal detection. Our dataset only included undisturbed sleep of healthy individuals screened for sleep disorders so that, aside for occasional hypopnoea/apnoea or movement, the vast majority of detected arousals occurred spontaneously. While the literature does not tell us whether spontaneous arousals may differ from induced arousals, our algorithm’s performance should be assessed in sleep-related pathologies (e.g., apnoea) to further enlarge its validation.

## Figures and Tables

**Table 1:** Distribution of recordings in the dataset across raters from Basel according to age and sex.

SCORER	YOUNG			OLDER			
	F	M	Total	F	M	Total	
BAS1	3	3	6	4	5	9	**15**
BAS2	0	4	4	2	2	4	**8**
BAS3	2	2	4	0	1	1	**5**
BAS4	2	2	4	2	1	3	**7**
TOTAL	7	11	18	8	9	17	**35**

**Table 2:** Cohen’s κ values and their interpretation, from [38].

Kappa Value	Interpretation
<0.00	Poor
0.00–0.20	Slight
0.21–40	Fair
41–0.60	Moderate
0.61–0.80	Substantial
0.81–1.00	Almost perfect

**Table 3:** Agreement coefficients (mean and standard deviation) between HR, with each HR being compared in turn to the other considered as gold standard: S_s_ (inter-rater agreement); κ (Cohen’s kappa); S_e_ (sensitivity); C_s_ (mean overlap of events); FDR_e_ (false discovery ratio).

Gold Standard	Compared	S_s_	κ_s_	Se_e_	C_s_	FDR_e_
BAS	DC	94 ± 3%	0.97 ± 0.02	58 ± 16%	72 ± 7%	36 ± 12%
DC	BAS	89 ± 4%	0.94 ± 0.02	81 ± 26%	78 ± 12%	78 ± 9%

**Table 4:** Agreement coefficients (mean ± standard deviation) for all recordings between AD and HR scoring, for both HR inclusive and HR conservative detection as gold standard. EMG indicates automatically detected events that are accompanied by a submental EMG tone increase.

Gold Standard	S_s_	κ_s_	Se_e_	C_s_	FDR_e_
HR inclusive EMG	86 ± 6% 90 ± 6%	0.93 ± 0.03 0.95 ± 0.03	67 ± 23% 45 ± 23%	59 ± 13% 61 ± 15%	61 ± 16% 28 ± 25%
HR conservative EMG	88 ± 4% 92 ± 4%	0.94 ± 0.02 0.96 ± 0.02	83 ± 26% 64 ± 27%	58 ± 14% 60 ± 15%	74 ± 12% 41 ± 23%

**Table 5:** Raw number of arousals detected per rater for young and older individuals (mean ± SD).

	DC	BAS *	Inclusive	Conservative	AD	AD EMG
Young	63 ± 31	84 ± 30	93 ± 37	51 ± 19	208 ± 48	68 ± 22
Old	79 ± 34	141 ± 53	142 ± 46	67 ± 23	193 ± 39	70 ± 25

* significant difference between age groups (*p* = 0.0006).

**Table 6:** Results of GLMMs for each agreement coefficient and age/sex for Automatic Detection (AD) against HR inclusive and conservative detection as gold standard, with all AD events (normal) and only AD EMG-associated events (italic).

Gold Standard	AD Arousals		S_s_	κ_s_	Se_e_	C_s_	FDR_e_
HR inclusive	All	Age Sex	*p* = 0.10	*p* = 0.12	***p* = 0.01**	*p* = 0.24	***p* = 0.05**
			F = 2.83	F = 2.49	**F = 7.53**	F = 1.42	**F = 4.23**
			*p* = 0.10	*p* = 0.10	*p* = 0.16	*p* = 0.21	*p* = 0.49
			F = 2.91	F = 2.83	F = 2.12	F = 1.63	F = 0.48
	*EMG*	*Age Sex*	p = 0.002 *	p = 0.002 *	***p = 0.01***	*p = 0.41*	*p = 0.61*
			***F = 11.48***	***F = 11.03***	***F = 6.89***	*F = 0.71*	*F = 0.26*
			*p = 0.07*	*p = 0.07*	*p = 0.78*	*p = 0.13*	*p = 0.70*
			*F = 3.49*	*F = 3.48*	*F = 0.08*	*F = 2.41*	*F = 0.15*
HR conservative	All	Age Sex	*p* = 0.96	*p* = 0.91	*p* = 0.33	*p* = 0.86	*p* = 0.07
			F = 0.00	F = 0.01	F = 0.99	F = 0.03	F = 3.52
			*p* = 0.21	*p* = 0.21	*p* = 0.12	*p* = 0.27	*p* = 0.37
			F = 1.64	F = 1.62	F = 2.49	F = 1.24	F = 0.82
	*EMG*	*Age Sex*	*p = 0.09*	*p = 0.10*	*p = 0.05*	*p = 0.97*	*p = 0.88*
			*F = 3.05*	*F = 2.87*	*F = 4.21*	*F = 0.00*	*F = 0.02*
			***p = 0.04***	***p = 0.04***	*p = 0.45*	*p = 0.21*	*p = 0.56*
			***F = 4.68***	***F = 4.71***	*F = 0.58*	*F = 1.63*	*F = 0.35*

All F tests had 1 (main effect) and 32 (error) degrees of freedom. * Significant after correcting for multiple comparisons (*p* < 0.005—Bonferroni corrected).

## Code Availability

The described algorithm is freely available on gitlab at https://gitlab.uliege.be/CyclotronResearchCentre/Public/fasst. It runs using FASST, a M/EEG toolbox developed by researchers from the Cyclotron Research Centre, University of Liège, Belgium, with the financial support of the Fonds de la Recherche Scientifique-FNRS, the Queen Elizabeth’s funding, and the University of Liège (Leclercq et al., 2011). The software FASST and its manual are available at http://www.montefiore.ulg.ac.be/~phillips/FASST.html.

## Abbreviations

AD	Automatic detection
EEG	Electroencephalogram
EMG	Electromyogram
FDR	False discovery ratio
HR	Human rater
SD	Standard deviation
